# Novel Clinical and Diagnostic Aspects of Antineutrophil Cytoplasmic Antibodies

**DOI:** 10.1155/2014/185416

**Published:** 2014-06-05

**Authors:** Johannes Schulte-Pelkum, Antonella Radice, Gary L. Norman, Marcos Lόpez Hoyos, Gabriella Lakos, Carol Buchner, Lucile Musset, Makoto Miyara, Laura Stinton, Michael Mahler

**Affiliations:** ^1^Department of Research, INOVA Diagnostics Inc., 9900 Old Grove Road, San Diego, CA 92131-1638, USA; ^2^Microbiology and Virology Institute, San Carlo Borromeo Hospital, Via Pio II 3, 20153 Milan, Italy; ^3^Division of Immunology, University Hospital Marqués de Valdecilla-IFIMAV, Avenida Valdecilla, 39008 Santander, Spain; ^4^Department of Immunology and Internal Medicine, Hospital of Pitie-Salpêtriere, Boulevard de l'Hôpital, 75634 Paris, France; ^5^Department of Medicine, University of Calgary, 3330 Hospital Dr. NW, Calgary, AB, Canada T2N 4N1

## Abstract

Antineutrophil cytoplasmic antibodies (ANCA) are the serological hallmark of some idiopathic systemic vasculitides. Besides the investigation of ANCA-associated vasculitis (AAV) and constant effort for a standardized nomenclature and classification of the AAV, a main focus of research during the last few years has been to constantly improve the performance of enzyme immunoassays. With the latest so called third generation ELISA, this goal seemed to be fulfilled. The International Consensus Statement on Testing and Reporting of ANCA gave recommendations for standardized strategies for the serological diagnosis of ANCA. New developments now target the system immanent drawbacks of the respective diagnostic methods, be it the need for batching and the long time to result for ELISA, or the high likelihood of error and subjectivity of indirect immunofluorescence (IIF). Random access technology and multiplexing for solid phase assays as well as digital imaging for IIF are tools which may help to expedite and simplify routine diagnostics in the lab and in emergency settings. Recent findings indicate that PR3-ANCA have clinical utility beyond the diagnosis of AAV. PR3-ANCA can also serve as an aid for the differentiation between ulcerative colitis (UC) and Crohn's disease (CrD) and the stratification of UC patients. This review provides a detailed review of what is known about ANCA and highlights the latest research and state-of-the-art developments in this area.

## 1. Introduction

### 1.1. Historical Perspectives on Antineutrophil Cytoplasmic Antibodies (ANCA)

Antineutrophil cytoplasmic antibodies (ANCA) are directed against primary granules of neutrophils and are associated with neutrophil-mediated inflammation [1]. ANCA were first described in 1982 by Davies et al. in a series of patients with segmental necrotizing glomerulonephritis (FNGN) and symptoms of systemic vasculitis [2]. In 1985 van der Woude et al. reported the strong association of ANCA producing a diffuse granular cytoplasmic staining pattern on ethanol-fixed neutrophils (C-ANCA) and granulomatosis with polyangiitis (GPA) (formerly known as Wegener's granulomatosis (WG)) [3, 4]; a few years later, ANCA producing a perinuclear fluorescent pattern (P-ANCA) on the same cellular substrate were described in patients with idiopathic necrotizing crescentic glomerulonephritis and microscopic polyangiitis (MPA) [5]. Initially, the only method available for ANCA detection was the indirect immunofluorescence (IIF) test on normal human ethanol-fixed neutrophils [3]. Although currently numerous assay formats such as enzyme linked immunoassays (ELISA), chemiluminescent immunoassays (CLIA), lateral flow assays (LFA), and combinations of IIF and microbead assays have been made available, the IIF still often remains the method of choice for initial screening.

### 1.2. Terminology and Molecular Biology of ANCA

**Figure 1:**
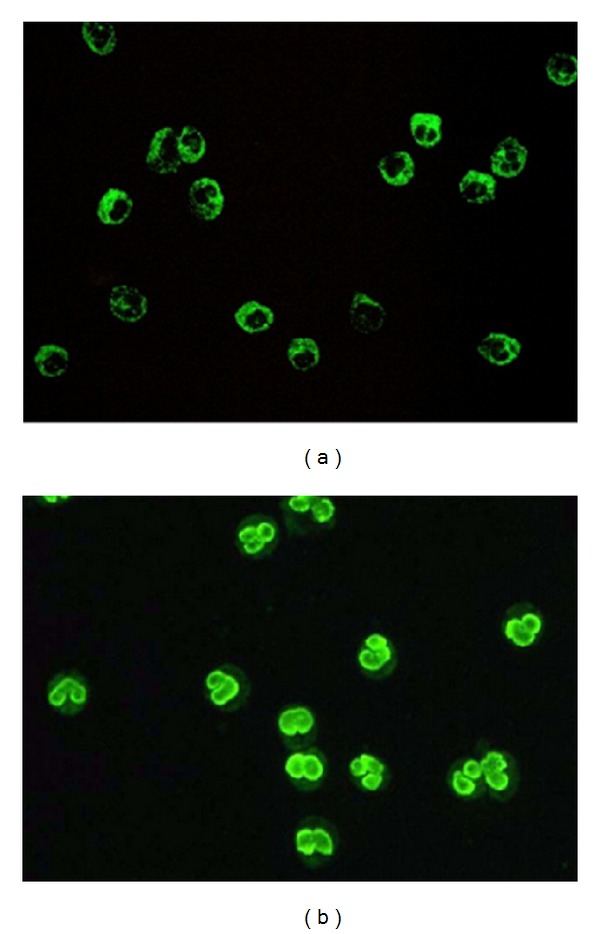
Appearance of cytoplasmic indirect immunofluorescence pattern (C-ANCA, Figure 1(a)) and perinuclear (P-ANCA, Figure 1(b)) on ethanol-fixed human neutrophil cells. The C-ANCA pattern is largely caused by autoantibodies targeting serine protease proteinase-3 (PR3-ANCA), while the P-ANCA pattern is caused by antibodies binding to many antigens among which myeloperoxidase (MPO-ANCA) is the most frequent target in primary systemic vasculitis [11].

The classical terms C-ANCA and P-ANCA describe IIF patterns on granulocyte substrates [5–7]. C-ANCA (Figure 1(a)) is largely due to the presence of autoantibodies targeting the serine protease proteinase-3 (PR3), while P-ANCA (Figure 1(b)) is caused by antibodies directed mainly against myeloperoxidase (MPO). Additionally, antinuclear antibodies (ANA) and antibodies against the cytoplasmic granule antigens lactoferrin, lysozyme, azurocidin, elastase, cathepsin G, bactericidal/permeability-increasing enzyme (BPI) show the so-called atypical ANCA pattern on ethanol-fixed neutrophils [8–10]. MPO is the most frequently recognized antigen in P-ANCA and primary systemic vasculitis [11]. PR3 is a weak cationic protein of 29-30 kDa molecular weight (MW), belonging to the trypsin family of serine proteases. PR3 is synthesized as a preproenzyme and subsequently processed in four steps into the mature form. It is stored in the azurophilic granules of neutrophils but can also be found within the membrane of secretory vesicles. PR3 is physiologically inhibited by *α*1-antitrypsin [12]. MPO, which represents about 5% of the total protein content of neutrophils, is a strong cationic molecule (isoelectric point > 11) made up of a heterodimer with a MW of ~140 kDa. The enzyme is characterized by a powerful bactericidal activity, whose peroxidase activity is physiologically inhibited by ceruloplasmin [13].

### 1.3. Clinical Utility of ANCA

**Figure 2:**
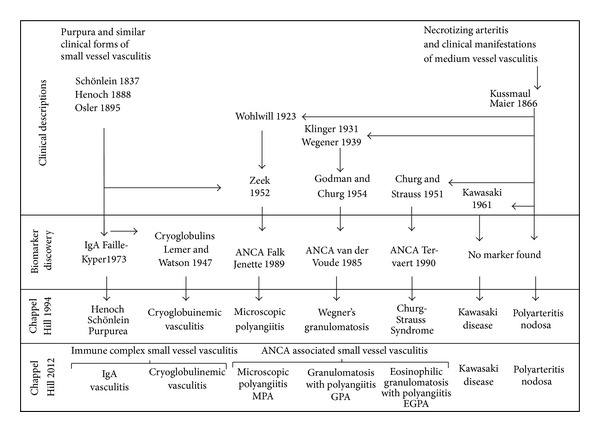
Disease nomenclature system adopted by the 2012 International Chapel Hill Consensus Conference [17].

ANCA are the serological hallmark of some idiopathic systemic vasculitides, and the term ANCA-associated vasculitis (AAV) has been used to collectively name those primary small vessel vasculitic syndromes in which circulating ANCA are commonly found: MPA and its renal limited form (pauci-immune necrotizing glomerulonephritis), GPA, and eosinophilic granulomatosis with polyangiitis (EGPA) [formerly known as Churg-Strauss syndrome (CSS)] [14, 15]. This approach, adopted by the International Chapel Hill Consensus Conference (CHCC) and by the European Vasculitis Study Group (EUVAS), is supported by the striking clinical and histological similarities between the AAV, the widespread use of ANCA as a diagnostic marker, and the growing evidence of their pathogenetic potential [16]. An overview of the previous and the new disease nomenclature as proposed by the 2012 CHCC for defining small vessel vasculitis [15] is shown in Figure 2.

Apart from AAV, ANCA are also useful in the diagnosis and classification of inflammatory bowel disease (IBD) [19, 20], autoimmune hepatitis (AIH), and primary sclerosing cholangitis (PSC) [21]. Here, the IIF usually produces a perinuclear pattern,with homogeneous or fine speckled cytoplasmic fluorescence on ethanol-fixed slides [22]. The term atypical ANCA has been proposed to describe this pattern [23].

### 1.4. Pathogenicity of ANCA

Since 1995* in vitro*,* in vivo* and clinical studies have been providing increasing evidence in favour of a pathogenetic role for ANCA (especially MPO-ANCA) in the development of AAV. However, to induce severe damage, ANCA have been shown to require additional triggers [24]. AAV are multifactorial diseases, and the involvement of genetic factors in disease pathogenesis is considered important as are environmental factors such as silica exposure, infections (in particular with* Staphylococcus aureus*) and treatment with propylthiouracil and other drugs. Discovered by Kain et al. in 1995, autoantibodies to LAMP-2, a suggested novel ANCA subtype, have been reported to be present in almost all patients with active, untreated AAV, and renal involvement [8, 25]. The authors also suggested a previous unrecognized molecular explanation for the origin and development of injury in* pauci-immune* GN; however, such findings could so far not be confirmed by a subsequent study [26]. Controversy exists about the true prevalence, pathogenicity, and practical utility of anti-LAMP-2-autoantibodies for the management of AAV patients. Another new aspect of the pathology of ANCA is the autoantibody stimulation by neutrophil extracellular traps (NETs), also known as NETosis [27]. NETs are formed from extracellular nuclear DNA of neutrophils released by a programmed cell death different to apoptosis, releasing strands of nuclear DNA spiked with antibacterial proteins [28]. Bacteria, viruses, and fungi are trapped and killed in these NETs, and besides antimicrobial proteins PR3 and MPO are present in the sticky DNA fibers [27–29]. Evidence of NETosis as a possible trigger of AAV is evolving, as it was shown that ANCA not only induce the neutrophil oxidative burst but also can induce the programmed release of NETs in absence of a microbial infection and, even more interesting, that the transfer of activated myeloid dendritic cells enriched with NET components into naive mice could cause AAV [30]. Also the ineffective clearance of NETs from the endothelial vessel wall could give clue to the disease progression in AAV.

## 2. Diagnostic Methods for ANCA Detection

### 2.1. Overview

The sensitive and specific detection of antibodies to PR3 and/or MPO is highly recommended in patients with suspected systemic vasculitis, occurring in about 80% of all AAV patients. Only fast and adequate treatment can avoid the development of organ failure [31, 32]. In 1999, the International Consensus Statement on Testing and Reporting of ANCA was published, which provided suggestions for ANCA testing and reporting [23]. An addendum to this statement was published in 2003 with the quality control guidelines, comments, and recommendations for ANCA testing in other autoimmune diseases [33]. According to these guidelines, the initial screening method for ANCA is IIF on ethanol-fixed neutrophils of human origin with confirmation by a solid phase assay (i.e., ELISA). The specificity of the individual tests (C-ANCA, P-ANCA, PR3, and MPO) is not satisfactory; however, combining IIF and an ELISA for PR3/MPO provides a specificity of 99% versus pathological controls, with only a minor loss of sensitivity [34].

The identification and purification of the PR3 and MPO antigens [5, 35, 36] allowed for the development of several immunoassays for the quantitative detection of antibodies specific for PR3 and MPO, including the conventional ELISA and, more recently, line immunoassays (LIA) [37], capture [38, 39], and anchor assays [40, 41] as well as multiplex assays [42–44]. As ELISAs are only moderately fast with assay times between 1.5 and 3 hours, the focus lately shifted towards a decrease in assay time and fully automated technologies. Lateral flow assays (LFA) reduce time to result and require limited laboratory equipment. To reduce time to result and minimize hands-on time in the laboratory, new systems combining random access and chemiluminescent immunoassay technology (CLIA) have been developed and offer single patient testing together with assay times of as little as 30 minutes [45].

### 2.2. Indirect Immunofluorescence for ANCA Detection

The International Consensus Statement on Testing and Reporting of ANCA recommends that the minimum requirements for ANCA testing are as follows: “IIF should be performed on all sera from new patients, since 10% of ANCA positive sera in patients with GPA or MPA can be demonstrated only by IIF” [23, 33]. In spite of this, the strategy for the detection of ANCA varies across laboratories, according to geographical areas, traditions, and local experience. Additionally, the IIF method in general, faces many challenges and occupies a special and often not very popular place in the laboratory. Historically both slide preparation and slide reading require considerable manual work, are time consuming, and are prone to technical problems in the hands of less experienced users [46–48]. Furthermore, the handwritten transcription of results in the dark room is a hotbed of transcription errors. High intra- and interlaboratory variability are also observed in ANCA testing by IIF due to differences in neutrophil sources, preparation, fixation, and subjectivity of laboratory technologists. Unique to ANCA by IIF is the recommendation for two different substrates (ethanol- and formalin-fixed neutrophils) and the frequent presence of cross-reactive ANA.

**Figure 3:**
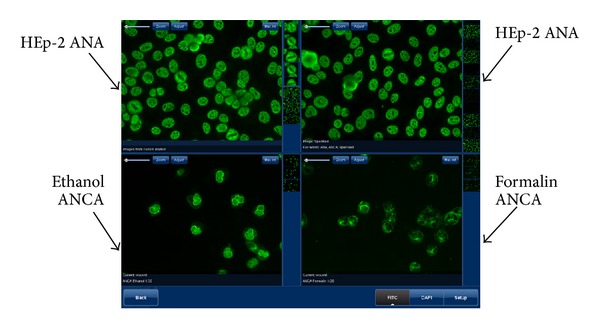
Representative screenshot of a Multianalyte Screen (i.e., QUANTA Link, INOVA Diagnostics Inc.) showing a C-ANCA and speckled ANA double positive sample. Images obtained on ethanol- and formalin-fixed substrates can be displayed next to Hep-2 ANA results simultaneously and thus allow for interpretation with high accuracy.

In recent years, automated fluorescent microscope systems that acquire, store, and display high resolution digital images obtained on IIF slides have been developed (NOVA View, INOVA Diagnostics Inc. USA; Aklides, Medipan GmbH, Germany; Image Navigator, Immuno Concepts Ltd., USA; EURO-Pattern, Euroimmun AG, Germany). Digital images can be viewed and used any time for follow-up, training, and consultation/second opinion purposes. Software programs provide tools to support the operator's decision making such as negativity, positivity, and pattern interpretation [46–50]. An important feature to increase the accuracy of IIF interpretation is the possibility of multianalyte screening and computerized display. Such systems can display results of the same sample on different substrates (i.e., results obtained on ethanol- and formalin-fixed substrates next to results obtained on HEp-2 ANA; see Figure 3). However, these programs should not be used to provide final results as they require competent human confirmation for analysis and reporting. This process does offer the benefits of reducing hands-on time and transcription errors in addition to eliminating the need for a dark room.

### 2.3. ELISA for ANCA Detection

After an initial IIF screen, the results should be verified by ELISA [23] or an equivalent solid phase assay. In the past, ELISA tests did not reach the sensitivity and specificity of IIF on neutrophils [51, 52], but, more recently, new methods offer improved results. These methods utilize different immobilization strategies resulting in presentation of the antigen in the most native way to enable the best exposure of all relevant epitopes to the antibody. Different immobilization techniques have led to advancement in the generation of immunoassays aimed to improve the sensitivity of the assays.

### 2.4. First Generation ELISA Tests

After the antigens targeted by autoantibodies generating the P-ANCA and C-ANCA pattern were described [5, 35, 36], the first ELISAs were developed using purified native PR3 and MPO antigens. These test kits used simple adsorption coating methods and the purity of the antigens was quite variable [7]. Most tests lacked comparability of results and correlation towards IIF methods [51]. The limited sensitivity of these first generation tests was attributed to the adsorption immobilization process resulting in masking and deformation of epitopes. In 1998, the International Consensus Statement on Testing and Reporting of ANCA by a proposed a combination of IIF and ELISA test methods [23].

### 2.5. Second and Third Generation Tests

The low sensitivity of the first generation ELISAs led to the development of novel approaches to protein modification and different assay methods. The so-called second generation ANCA tests used capture molecules, mostly antibodies, to bind the antigens to the surface without causing changes to the structure of the epitopes [53]. These ELISAs showed a significant increase in sensitivity and proved superior to the direct binding ANCA tests [53].

Third generation assays, again, aimed to prevent the antigen from being distorted, due to binding on the ELISA plate, utilize “anchor” techniques to immobilize the antigens [54]. The antigens are bound to the surface of the ELISA plate using anchor molecules which are attached to the surface of the ELISA plate. This method was discussed to provide a better accessibility of epitopes and thus in a further increase in the sensitivity and specificity as compared to IIF. A comparative ROC analysis of three generations of PR3-ANCA assays based on the detection of PR3-ANCA in the sera of 86 GPA patients and 80 healthy control and 450 disease control sera revealed AUC values of 0.80 [95% CI: 0.76–0.83] (1st generation), 0.86 [95% CI: 0.82–0.89] (2nd generation, capture ELISA), and 0.96 [95% CI: 0.94–0.98] (3rd generation Anchor ELISA), respectively [54].

### 2.6. Other ELISA Methods

A novel approach using direct coating in ELISA uses a mixture of a human native and human recombinant PR3 antigen [55]. This may offer a significantly higher sensitivity compared to first generation direct coating and second generation capture methods; however this has not been confirmed in all studies [56, 57].

### 2.7. Lateral Flow Assay Systems

Historically LFAs were regarded as fast, but insensitive, reaching only about 80% of the diagnostic performance of ELISAs [58–60]. Newer developments of LFA made use of liquid antigens and significantly improved the diagnostic performance of these tests. The use of liquid antigens improves the antigen-antibody binding. The antigens are added in solution, to minimize possible masking of epitopes due to surface adhesion. Interpretation of the test result can be done by a hand held reading device and results can be interpreted semi-quantitatively. Preliminary results indicate that this technology might reach the sensitivity of ELISAs with an assay time of 20 minutes [61, 62]. However, clinical use of LFA for the detection of ANCA is extremely limited at most.

### 2.8. Combination of IIF and Bead Assays

A new approach to combine screening for and confirmation of ANCA was recently presented. This new technology combines ethanol-fixed neutrophils and the antigens PR3, MPO, and GBM coupled to different microbeads which are then attached to different compartments of the IIF slide. The different reactivities can be identified by either the size of the respective bead or the position in different compartments of the IIF slides. A first validation of this assay comparing the results against a combination of IIF and ELISA results showed a positive agreement in 124/129 (96.1%) samples and negative agreement in 540/542 (99.6%) samples [63].

### 2.9. Chemiluminescent Immunoassays for ANCA

**Figure 4:**
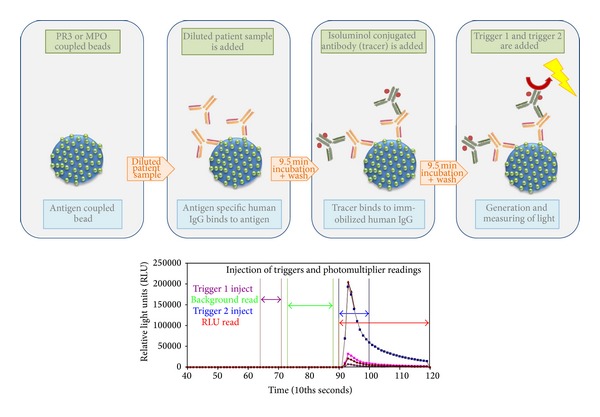
Principle of QUANTA-Flash chemiluminescent immunoassays. Paramagnetic beads are coupled with native PR3 or MPO. The beads are then incubated with diluted patient samples. After 9.5 min incubation unbound antibodies are removed by washing. Antihuman IgG isoluminol conjugate (Tracer) is added and binds immobilized antibodies. After another 9.5 min incubation unbound Tracer is removed by washing. Finally, Trigger 1 and Trigger 2 are injected and emerging light is measured. After injection of Trigger 1 and Trigger 2, the luminescence is measured as relative luminescence units (RLU) [17].

**Figure 5:**
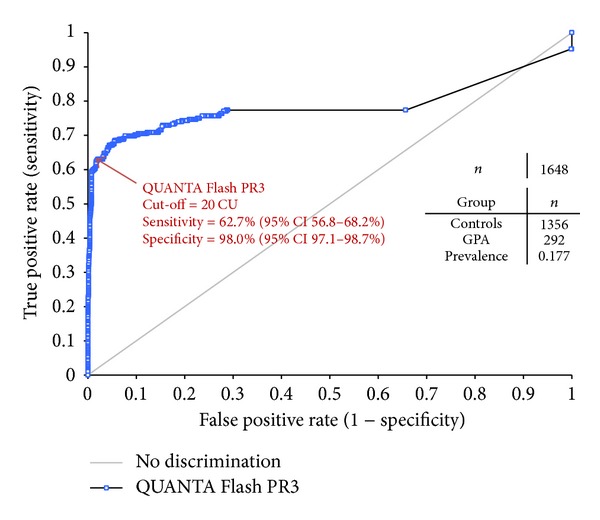
Receiver operation characteristics (ROC) analysis of 292 GPA serum samples tested together with 1356 disease controls using a novel PR3 CLIA revealed an area under the curve (AUC) value of 0.78 (95% CI 0.74–0.83), resulting in a clinical sensitivity and specificity of 62.7% (95% CI 56.8–68.2%) and 98.0% (95% CI 97.1–98.7%), respectively.

**Figure 6:**
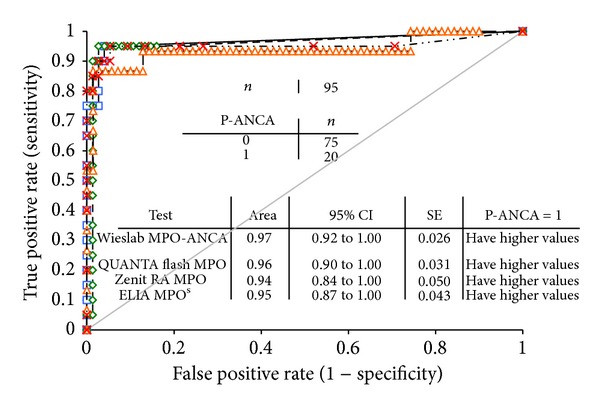
Comparative receiver operation characteristics (ROC) analysis of 20 P-ANCA positive serum samples tested by IIF together with 75 disease and control serum samples using a novel MPO CLIA and three other commercially available MPO assays revealed AUC values of 0.97 (Wieslab MPO-ANCA), 0.96 (QUANTA Flash MPO), 0.94 (Zenit RA MPO), and 0.94 (ELIA MPO^s^) resulting in a relative sensitivity/specificity of 95%/96% (Wieslab MPO), 95%/96% (QUANTA Flash MPO), 86.7%/95.7% (Zenit RA MPO), and 95%/94.7% (ELIA MPO^s^) against a positive P-ANCA result by IFA, respectively [18].

Chemiluminescent assays (CLIA) are significantly different from ELISA technology, as the antigen is covalently attached to the surface of the bead particles unlike the passive adsorption used for most ELISAs. Almost at the same time, two different CLIA assays for the detection of PR3- and MPO-ANCA have been developed on two systems: Zenit RA (A. Menarini Diagnostica S.r.l., Florence, Italy) and BIO-FLASH (INOVA Diagnostics Inc., San Diego, USA). In the BIO-FLASH system the epitope conformation of native PR3 and MPO antigens and hence a reliable representation of epitopes have been reported [40, 53]. In CLIA, paramagnetic beads are coupled with native PR3 or MPO (Figure 4). After the beads are incubated with diluted patient serum and washed, antihuman IgG isoluminol conjugate antibody “Tracer” is added. The conjugate is oxidized when sodium hydroxide and peroxide solutions “Triggers” are added and the flash of light produced from this reaction is measured as relative light units (RLUs). The RLUs are proportional to the amount of isoluminol conjugate that is bound to the human IgG, which is in turn proportional to the amount of autoantibodies bound to the antigen on the beads. Unlike ELISA or addressable laser bead immunoassays (ALBIA) platforms, the detection system uses a proprietary CLIA technology that affords a remarkably wider dynamic range, an entirely linear titration curve, and more consistent inter- and intratest reliability [64]. Recently the high sensitivity and specificity of a PR3 CLIA, for example, could be demonstrated in a large multicenter study covering 11 laboratory sites in 9 different countries [65]. A total of 1648 samples (292 GPA and 1356 disease controls) were tested using a new PR3 CLIA assay (QUANTA Flash PR3), which could discriminate GPA patient samples from various disease controls with a ROC AUC value of 0.78 and a specificity of 98.0% at a sensitivity of 62.7% (Figure 5). In a smaller part of this multicenter study a comparison against another assay revealed high percentages of agreement between the new CLIA assay and another well-established high sensitivity PR3 Assay (ELIA PR3^s^ Thermo Scientific, Germany) (Table 1). Both assays could discriminate between samples from GPA patients and controls with similar sensitivities and specificities: 56.1% (95% CI 44.7–67.0%)/98.2% (95% CI 93.8–99.8%) sensitivity/specificity for QUANTA Flash, and 58.5% (95% CI 47.1–69.3%)/96.5% (95% CI 91.3–99.4%) sensitivity/specificity for ELIA PR3^s^. In another study 95 samples (20 P-ANCA positive by IIF versus 75 controls) were tested with a newly developed MPO CLIA in comparison to three different commercially available MPO-assays (Wieslab MPO ANCA ELISA, Eurodiagnostica AB Sweden; Zenit RA MPO ELISA, A. Menarini Diagnostica S.r.l., Florence, Italy; and ELIA MPO^s^, Thermo Scientific, Germany). Both the MPO CLIA and the Wieslab MPO-ANCA ELISA revealed highest relative sensitivity/specificity compared to a positive p-ANCA IIF result (Figure 6) reflected by AUC values of 0.97 (95% CI 0.92–1.00) for the Wieslab MPO-ANCA ELISA and 0.96 (95% CI 0.90–1.00) for the MPO CLIA [18].

### 2.10. Summary of Methods for ANCA Detection

Despite several comparative studies, it remains debatable as to which methodology for ANCA detection provides the highest clinical accuracy for the diagnosis of SVV [53, 65, 66]. Several studies published over the last decade suggested that the sensitivity of both capture as well as novel anchor assays were superior to classical ELISA and even to IIF [40, 41, 53, 67, 68]. Lately the new emerging technology of CLIA showed comparable or superior sensitivity compared to established assay types.

### 2.11. ANCA Testing in Emergency Setting

ANCA-associated vasculitides are chronic multisystemic disorders, affecting several organs, and characterized by the occurrence of flares and remissions. Because of severe manifestations including alveolar haemorrhage, rapid progressive glomerulonephritis, scleritis, and necrotizing sinusitis, it is of the utmost importance to propose ANCA results in a timely manner [31, 32]. ANCA testing at presentation and in the case of suspected disease flares should be requested urgently, in order to rapidly initiate appropriate immunosuppressive therapies and avoid irreversible organ damages [68, 69].

Relapses of the diseases occur in 30–60% of the patients during 5–10 years of follow up and increase the risk of* End Stage Renal Disease* (*ESRD*); therefore ANCA testing is also required for the follow-up of the patients [70, 71]. According to disease stage and activity, follow-up assessments include ANCA testing at intervals of several months (three to six months). Even if serial ANCA testing is controversial, ANCA testing is usually ordered in case of suspicion of relapse, side effect of immunosuppressive therapy or for differential diagnosis with undercurrent infectious diseases [72, 73]. IIF is inappropriate in the emergency setting due to its time constraints and inability to quantify results.

Although controversially discussed, ANCA testing at intervals is recommended as ANCA titres may decline during effective treatment and often rise prior to relapse [21, 74, 75]. In contrast to IIF, tests that give quantitative ANCA measurements are required to monitor disease activity. Lately it was revealed that the use of native PR3 antigen together with new assay technologies led to results correlating better to the Birmingham Vasculitis Activity Score (BVAS) than assays using a combination of recombinant and native PR3 antigen [45, 56].

## 3. ANCA as Biomarkers in Inflammatory Bowel Disease

Atypical P-ANCA is found in patients with inflammatory bowel disease (IBD), mainly ulcerative colitis (UC) [76]. When combined with anti-*Saccharomyces cerevisiae* antibodies (ASCA), atypical P-ANCA has been recommended as a way to help distinguish UC from Crohn's disease (CrD) [22, 77–81]. ASCA seropositivity is a predominant feature of CrD, while atypical P-ANCA is a marker of UC (Table 2) [22, 78]. Both ASCA and ANCA have been reported to predict the development of IBD [82]. Despite several studies, the specificity of ANCA in IBD remains poorly defined [77, 83]. The diagnosis of IBD including UC and CrD is largely based on endoscopic and histological assessment of the inflamed tissue [77, 84]. While several antibody tests can assist in the diagnosis of CrD including ASCA and pancreatic zymogen granule protein 2 (GP2) [85–87], the only serological biomarker for UC is atypical P-ANCA detected by IIF [22]. However, IIF has the limitations described above and is unable to provide information about ANCA antigen specificity [77, 79]. Several studies have attempted to identify the major target antigen of atypical P-ANCA in IBD [83, 88], but major disease-specific target antigens are still missing.

In contrast to historical data [89, 90], more recent studies reported PR3-ANCA in a significant percentage of IBD patients [44]. This raises the possibility that akin to AAV, PR3-ANCA may also be a marker for IBD. However, early studies of PR3-ANCA in IBD have been based on relatively small cohorts of UC patients [44, 91, 92]. When twelve PR3 ANCA assays were compared using 22 IBD sera, the reported prevalence of PR3-ANCA ranged from 4% to 43%, raising concerns as to the reliability of the assays used in these studies [44, 91–93]. The two assays with the highest sensitivity [BINDAZYME (Binding Site Ltd.) sensitivity: 39% and Rainbow ELISA PR3 (Bio-diagnostics Ltd.) sensitivity: 43%] also showed the lowest specificity (88%) [44].

Recently it was suggested that PR3-ANCA measured by a novel chemiluminescent immunoassay (CLIA) on a random access autoanalyzer (BIO-FLASH) are useful in the differential diagnosis of UC and CrD [19]. Our observation that PR3-ANCA can be detected in sera from patients with IBD, with higher prevalence in UC versus CrD patients suggests that PR3-ANCA testing could assist in discriminating UC from CrD and in discriminating IBD from other gastrointestinal conditions. The terms “indeterminate colitis” or “IBD unclassified” (IBD-U) categorize patients in whom the diagnosis of UC or CrD is not clear [77, 94, 95]. The differential diagnosis may be complicated in patients with irritable bowel syndrome, celiac disease, or other gastrointestinal diseases with symptoms indistinguishable from those seen in IBD [77]. Furthermore, our data pointed to a possible role as biomarker for a lesser need of immunomodulatory therapy in UC patients with PR3-ANCA [19], although these data should be confirmed in further studies.

### 3.1. Differentiation between PR3-ANCA Positive UC and GPA Patients

**Figure 7:**
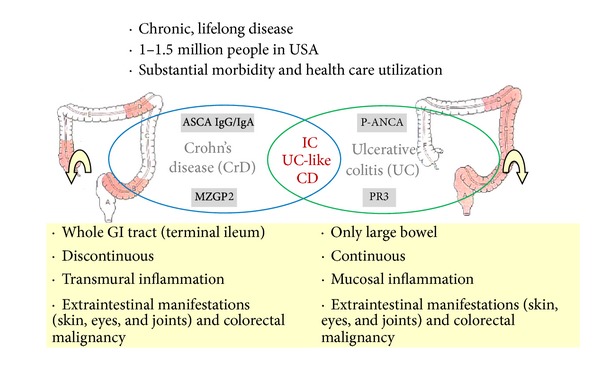
Differentiation between ulcerative colitis (UC) and Crohn's disease (CrD). The clinical difference of UC and CrD and the potential role of PR3-ANCA as a marker to help in the diagnosis of UC are illustrated.

The fact that PR3-ANCA is found in UC could be interpreted as compromising the specificity of PR3-ANCA for GPA. However, PR3-ANCA in patients with GPA are often associated with a C-ANCA pattern on ethanol-fixed neutrophils, while in UC patients an atypical P-ANCA is most often observed. The latter is most likely explained by reactivity to other antigens that have been reported in the past to be associated with UC [20]; see Figure 7. Consequently, differential diagnosis of GPA and UC in PR3-ANCA positive individuals can be achieved using the clinical presentation of the patient and the combination of PR3-ANCA and IIF.

Interestingly recent studies have described patients with overlapping features of UC and GPA [96–98]. To what extent PR3-ANCA positive UC patients will develop full-blown AAV over the course of their disease needs to be assessed in large longitudinal studies. It is widely appreciated that different autoimmune diseases can overlap in certain patients, which was recently described as polyautoimmunity [99]. Therefore, additional studies are required to determine whether there is an overlap between the two chronic inflammatory diseases. While GPA typically affects the upper respiratory tract and the kidneys, UC is limited to the colon. Although, 10% of patients with SVV, can present with ulcerations of the colon [100], isolated gastrointestinal tract involvement is infrequently seen in ANCA-positive patients with SVV [101].

## 4. Conclusion 

The names of the common forms of vasculitis have been recently revised so that the eponyms such as WG and CSS have been changed to GPA and EGPA, respectively. ANCA testing by IIF remains an important first step when screening for ANCA. Positive results, or cases of strong clinical suspicion should be run on a solid phase assay for confirmation of the antigenic specificity and quantitation of the results. Automated solutions for autoimmune laboratories performing IIF assays simplify and streamline the IIF reading/interpretation workflow and increase the reliability of IIF testing by sample traceability. Despite big improvements in sensitivity and specificity, ELISA methods often are burdened with batching and relatively long result times. Acute disease flares can be confounding, so accurate, rapid diagnosis followed by appropriate therapy is paramount to halting the deleterious effects of ANCA vasculitic disease, especially in an emergency setting. Not all PR3 assays are suited for disease activity monitoring. PR3-ANCA are present in a significant percentage of IBD patients and may be a marker of concurrent SVV related disease. PR3-ANCA measured by CLIA are promising to aid in the differential diagnosis of UC and CrD.

## Figures and Tables

**Table 1:** Agreement between a new PR3 CLIA assay and an established high sensitivity PR3 assay. Spearman's rho of results was found at 0.74 (95% CI 0.67–0.80, data not shown) [65].

All patients (*n* = 196)	ELIA PR3^s^			Percent agreement (95% confidence)
	Positive	Negative	Total	
PR3 CLIA				
Positive	45	3	**48**	Pos. Agree = 86.5 (74.2–94.4%)
Negative	7	141	**148**	Neg. Agree = 97.9 (94.0–99.6%)
Total	**52**	**144**	**196**	Total Agree = 94.9 (90.8–97.5%)

**Table 2:** Prevalence of autoantibodies in ulcerative colitis and Crohn's disease [22, 77, 78, 82, 85].

Marker	Ulcerative colitis	Crohn's disease
P-ANCA	40–60%	5–20%
ASCA IgG	5–10%	40–70%
ASCA IgA	5–10%	40–70%
PR3-ANCA	15–40%	0–10%
MZGP2	0–5%	20–40%
